# The Relationship between Life Course Socioeconomic Conditions and Objective and Subjective Memory in Older Age

**DOI:** 10.3390/brainsci11010061

**Published:** 2021-01-06

**Authors:** Morgane Künzi, Emilie Joly-Burra, Sascha Zuber, Maximilian Haas, Doriana Tinello, Chloé Da Silva Coelho, Alexandra Hering, Andreas Ihle, Gianvito Laera, Greta Mikneviciute, Silvia Stringhini, Bogdan Draganski, Matthias Kliegel, Nicola Ballhausen

**Affiliations:** 1 Cognitive Aging Lab (CAL), Faculty of Psychology and Educational Sciences, University of Geneva, Boulevard du Pont d’Arve 28, 1205 Geneva, Switzerland; Emilie.Joly@unige.ch (E.J.-B.); Sascha.Zuber@unige.ch (S.Z.); Maximilian.Haas@unige.ch (M.H.); Doriana.Tinello@unige.ch (D.T.); Chloe.DaSilvaCoelho@unige.ch (C.D.S.C.); Gianvito.Laera@unige.ch (G.L.); Greta.Mikneviciute@unige.ch (G.M.); Matthias.Kliegel@unige.ch (M.K.); 2 Centre for the Interdisciplinary Study of Gerontology and Vulnerability (CIGEV), University of Geneva, Boulevard du Pont d’Arve 28, 1205 Geneva, Switzerland; A.Hering@tilburguniversity.edu (A.H.); Andreas.Ihle@unige.ch (A.I.); N.M.Ballhausen@tilburguniversity.edu (N.B.); 3 LIVES, Overcoming Vulnerability: Life Course Perspective, Swiss National Centre of Competence in Research, University of Lausanne, Géopolis Building, 1015 Lausanne, Switzerland; 4 Department of Developmental Psychology, Tilburg School of Social and Behavioral Sciences, Tilburg University, Simon Building, Warandelaan 2, 5037 AB Tilburg, The Netherlands; 5 Center for Primary Care and Public Health (Unisanté), University of Lausanne, Rue du Bugnon 44, 1011 Lausanne, Switzerland; Silvia.Stringhini@hcuge.ch; 6 Unit of Population Epidemiology, Division of Primary Care, Geneva University Hospitals, Rue Gabrielle-Perret-Gentil 4, 1205 Geneva, Switzerland; 7 Laboratory of Research in Neuroimaging (LREN), Department of Clinical Neuroscience, Lausanne University Hospital, University of Lausanne, Champ de l’Air Building, Rue du Bugnon 21, 1011 Lausanne, Switzerland; Bogdan.Draganski@chuv.ch; 8 Neurology Department, Max Planck Institute for Human Cognitive and Brain Sciences, Stephanstrasse 1A, D-04103 Leipzig, Germany

**Keywords:** life course, socioeconomic conditions, prospective memory, subjective memory complaints, aging

## Abstract

While objective memory performance in older adults was primarily shown to be affected by education as indicator of life course socioeconomic conditions, other life course socioeconomic conditions seem to relate to subjective memory complaints. However, studies differ in which life course stages were investigated. Moreover, studies have explored these effects in an isolated way, but have not yet investigated their unique effect when considering several stages of the life course simultaneously. This study, therefore, examined the respective influence of socioeconomic conditions from childhood up to late-life on prospective memory (PM) performance as an objective indicator of everyday memory as well as on subjective memory complaints (SMC) in older age using structural equation modeling. Data came from two waves of the Vivre-Leben-Vivere aging study (n=993, $M_{age}=80.56$). The results indicate that only socioeconomic conditions in adulthood significantly predicted late-life PM performance. PM performance was also predicted by age and self-rated health. In contrast, SMC in older age were not predicted by socioeconomic conditions at any stage of the life course but were predicted by level of depression. In line with the cognitive reserve hypothesis, present results highlight the significance of education and occupation (adulthood socioeconomic conditions) for cognitive functioning in later life.

## 1. Introduction

A growing concern related to aging is the loss of cognitive abilities, especially memory [1,2]. Therefore, a central goal of cognitive aging research has been to identify the factors that affect cognitive functioning and impairments in older age. For this purpose, an increasing number of studies have started to explore the role of socioeconomic conditions in childhood, across adulthood, or in late-life for interindividual differences in middle-aged and older adults’ cognitive functioning (e.g., [3,4]). In detail, low socioeconomic status or indicators of disadvantageous socioeconomic conditions across the life course, such as low parental socioeconomic conditions in childhood (e.g., [5,6]), low level of education [7,8,9] or low income [8], have a negative impact on late-life cognitive performance and are associated with cognitive decline in aging (but also see [5,10,11]). Conceptually, one explanation may be that favorable life course socioeconomic conditions are thought to be associated with more stimulating activities and thus being involved in building up of cognitive reserve throughout the lifespan [12,13]. This reserve could lead to better health in later life [14] and particularly, through structural and functional brain differences, buffer against cognitive impairments and early cognitive decline [5,10,11]. Indeed, different studies show that indicators of socioeconomic conditions are related to structural brain change (e.g., hippocampus volume and frontal cortex) as well as functional brain activity (e.g., differing activation in the network of prefrontal, frontal, hippocampus and parietal working memory areas), showing up at different life course stages and various cognitive functions (i.e., executive function, memory and language) [15,16]. A study investigating the link between socioeconomic status and structural brain change has demonstrated that non-demented older adults with higher socioeconomic status are characterized by reduced brain volume and an accelerated volume loss. This study supports the cognitive reserve hypothesis in an anatomical way in suggesting that individuals with higher socioeconomic status have higher cognitive reserve that allow them to cope with brain pathology longer before its clinical expression [17].

Importantly, one key open issue in this literature is whether the socioeconomic conditions at different phases of the life course affect late-life cognition independently (i.e., the way it was examined by previous studies) or in combination with each other by carry-over effects (i.e., as most theories would suggest, e.g., cumulative disadvantage theory, see [18]). For instance, by providing access to the required qualifications, education can be accountable for occupation and the income of an individual [19,20]. A high level of occupation and thus a higher income, in turn, seems to be important for maintaining good health in later life [19]. Furthermore, not only childhood and adulthood socioeconomic position but also a change in socioeconomic position between childhood and adulthood (social mobility) have been shown to influence older adults’ cognitive functioning, with an upward mobility leading to better cognitive performance [21]. However, most studies so far only focused on the impact of one stage (particularly at childhood) of the socioeconomic conditions on cognition [5,6,22,23,24], rather than examining their interplay across the life course, or only focused on specific indicators of socioeconomic conditions [7,8,9,25,26]. Thus, the first aim of the present study was to systematically target and disentangle the unique effects as well as the interplay of socioeconomic conditions across childhood, middle adulthood and later life on older adult’s cognitive functioning.

A second important aspect of this study concerns the domains of cognitive functioning that are targeted. Thus far, most research has focused on either global cognition using broad clinical neuropsychological tests such as the Mini-Mental State Examination (MMSE) (e.g., [4,25]), or on specific cognitive domains using classical laboratory measures, such as retrospective memory tests (immediate and delayed recall), verbal fluency tests and the trail-making-test for speed, attention and cognitive flexibility [3,5,6,7,14,24]. Aiming to go beyond these classical laboratory measures, the current study focused on a cognitive domain that is particularly relevant in everyday life, which is prospective memory (PM [27,28]). PM, also called memory for delayed intentions, is a memory function that is highly involved in numerous daily activities such as remembering to take one’s medication, making it an essential predictor of functional independence [27,29,30]. In terms of age effects, PM was shown to decline in older age (e.g., [31,32]). Moreover, PM tasks seem to be sensitive to early stages of dementia (e.g., [33]), with worse PM performance already showing up at the early stage of dementia compared to healthy controls [34].

In the current study, in addition to an objective measure of memory test performance, subjective memory complaints (SMC) were targeted, as they constitute a common health-related worry in older adults [35,36]. Moreover, SMC have been shown to cause daily functioning impairment and are associated with the onset of Alzheimer’s disease [37,38]. Although objective and subjective memory are two important components of memory functioning in older age, subjective memory decline can also be reported without objectively observing a decline in memory [33]. Similarly, in the context of PM, objective PM performance and SMC were shown to be independent [39], and objective and subjective PM performance is only associated in individuals with relatively few SMC [40]. However, older individuals with subjective, but not objective, memory complaints performed worse than healthy controls on more naturalistic PM tasks (e.g., [41]), demonstrating that SMC provide additional information beyond objective PM performance. Therefore, the second aim of the current study was to explore the specific impact of life course socioeconomic conditions on PM and SMC, respectively. However, what is known about the relationship between socioeconomic conditions and PM or SMC?

To date, there are few studies that have looked at those questions. These studies seem to agree on the finding that lower education and lower occupation are associated with lower PM performance in older age [42,43] (see also [44]). However, no study up to date investigated the effects of childhood socioeconomic conditions like parental’s education or occupation on late-life PM, nor was the effect of late-life factors such as income explored. Regarding SMC, lower and middle socioeconomic status in childhood has been shown to increase the prevalence of late-life SMC [45]. However, results are mixed with respect to education and range from a positive correlation between years of education and SMC to no correlation to a negative one, i.e., fewer years of education predicting a higher level of SMC in older age [35,46,47,48]. However, here, the level of education has been found to affect the type of SMC, as well as strategies adopted to prevent forgetting [46]. As for PM, the effects of late-life socioeconomic conditions on SMC have not yet been investigated. Besides, in contrast to objective PM performance, SMC have been more strongly associated with personality (e.g., higher level of neuroticism and lower level of consciousness) and affective factors (e.g., depressive symptoms) [39,47,48,49,50]. Taken together, these results suggest that life course socioeconomic conditions might impact PM and SMC differently.

To sum up, evidence of the effect of socioeconomic conditions on both PM and SMC is scarce and inconclusive, thus requiring further investigation. Moreover, so far no study has focused on the specific effect of different stages of the life course socioeconomic indicators on PM and SMC while considering several stages simultaneously, which was the aim of the present study. In terms of predictions, we expected: (1) childhood socioeconomic conditions to predict adulthood socioeconomic conditions and adulthood socioeconomic conditions to predict late-life socioeconomic conditions (a carry-over effect of socioeconomic conditions throughout the life course); and (2) disadvantageous socioeconomic conditions over the life course to predict lower PM performance and higher SMC in older age. Given that measures of objective PM and SMC appear independent, (3) we further expected them to be predicted by different factors and thus aimed to explore which specific life course socioeconomic conditions predict objective and subjective memory of older adults.

## 2. Methods

### 2.1. Participants

In this study, data stem from the Swiss longitudinal interdisciplinary study Vivre-Leben-Vivere (VLV). The first wave of this study (VLV1) started in 2011 and included 3080 participants coming from five Swiss cantons (Basel, Bern, Geneva, Ticino and Valais). In the second wave (VLV2), which started in 2017, the same participants of VLV1 were recontacted except from people whose data were assessed via proxies, those who lived in the Italian-speaking area (Ticino canton) and those who were no longer retrieved. Further reasons for non-participation were restricted health status of the participants, refusal, they had passed away or they had not fully completing the interview due to their MMSE score lower than 21. The final sample of VLV2 included 1059 participants, of whom 993 participants fully completed the face-to-face interview that included the PM task (i.e., one of the two main outcomes of the present study). Participants in VLV2 were aged from 70.2 to 102.6 years, $M_{age}=80.56$, $SD_{age}=6.56$; 489 were women (49.24%). All participants gave their written informed consent before they participated in the study. The present study was conducted in accordance with the Declaration of Helsinki, and the protocol was approved by the ethics commission of the Faculty of Psychology and Social Sciences of the University of Geneva (project identification codes: CE_FPSE_14.10.2010 and CE_FPSE_05.04.2017). In return for their participation in the study, participants received a CHF 20 supermarket voucher or could decide to make a CHF 20 donation to an association offering services for older adults in Switzerland.

### 2.2. Procedure

Participants were sent a paper-and-pencil self-administered questionnaire and had to fill out the questionnaire before the face-to-face interviews. Face-to-face interviews including cognitive testings took place at the participant’s home (95% of the sample) or another convenient location (e.g., side room of a café), which was validated by the experimenter as being quiet and without disturbances. The first wave of the study (VLV1) was conducted by trained researchers, while the second wave (VLV2) was conducted by employees of a research institute (LINK institute) that were previously trained by psychologists.

### 2.3. Material

Participants of both waves of the VLV panel study completed numerous questionnaires and cognitive testings from multiple disciplines (e.g., psychology, sociology and socioeconomics, see also [51], for a complete description). In the following, only the tests (i.e., PM test, conducted in VLV2) and questionnaires (conducted both in VLV1 and VLV2) that are relevant for our research question are described.

#### 2.3.1. PM Task

To measure PM as an indicator of objective everyday memory functioning, in VLV2, four different tasks were instructed before the interview started. These tasks asked the participants to remember to execute different actions during the interview session (for similar procedure, see [52,53,54,55]). One point was attributed when a task was succeeded and zero when the participant failed to remember to perform the instructed task. Participants had to remember to repeat the words “red pencil” whenever the experimenter mentioned a red pencil (“red pencil task”), to remember to knock on the table twice, whenever questions about physical activity were addressed (“knock on table task”), to remember to mention their date of birth when being asked for their years of education (“date of a birth task”) and to remember to remind the experimenter to turn on his/her mobile phone at the end of the interview (“telephone task”). These tasks have the advantage to resemble laboratory tasks, but their ecological validity is higher than traditional paradigms.

#### 2.3.2. SMC Measure

To measure SMC, participants in VLV2 had to rate on a scale from 0 = ‘never’ to 3 = ‘always’ whether, in their everyday life, their memory would play tricks on them.

#### 2.3.3. Socioeconomic Conditions Throughout the Life Course

Different socioeconomic conditions were assessed at each phase of the life course (childhood, adulthood and late-life). Note that, for each indicator, a higher score reflects more disadvantageous socioeconomic conditions.

#### 2.3.4. Childhood Socioeconomic Conditions

Three items referring to childhood socioeconomic conditions were included in the self-administered paper-and-pencil questionnaire of VLV2. One item assessed the father’s occupation and two items assessed the education of the mother and father (for same procedure, see [3,14]). To indicate the father’s occupation, the Swiss socio-professional categories of the Swiss Federal Statistical Office (2017) (https://www.bfs.admin.ch/bfs/de/home/statistiken/arbeit-erwerb/nomenclaturen/spk2010.assetdetail.4082538.html) were used (coded from 0 for the highest to 10 for the lowest occupation category), and parental education equally was assessed based on the educational categories suggested by the Swiss Federal Statistical Office (2020) (https://www.bfs.admin.ch/bfs/de/home/statistiken/bildung-wissenschaft/bildungsindikatoren/themen/wirkung/bildungsstand.html) (coded from 0 for the highest to 6 for the lowest level of education).

#### 2.3.5. Adulthood Socioeconomic Conditions

Three items referring to adulthood socioeconomic conditions were assessed in VLV1 with two items on the participant’s occupation (first and last, coded from 0 for the highest to 10 for the lowest occupation category) and one item on the highest participant’s educational level attained (coded from 0 for the highest to 5 for the lowest level of education). Similar to parental education and father’s occupation, the participant’s education and occupation were assessed with the help of categories provided by the Swiss Federal Statistical Office (2017; 2020).

#### 2.3.6. Late-Life Socioeconomic Conditions

Three items, namely monthly income (coded from 0 for the highest to 8 for the lowest monthly income categories), making ends meet based on monthly income (coded from 0 for ease to 3 for difficulty in making ends meet) and household fortune (coded from 0 for the highest to 5 for the lowest household fortune categories), were used to assess current (i.e., at VLV2) late-life socioeconomic conditions.

#### 2.3.7. Missing Data and Missing-Data-Relevant Variables

Missing data were present in late-life socioeconomic indicators (7.55%, 3.22% and 13.08% missing for monthly income, making ends meet and household fortune, respectively). As preliminary analyses revealed missing data were not missing completely at random ($\chi^{2}\left(1602,N=993\right)=1688.73,p=0.07$), three variables related to late-life socioeconomic conditions (i.e., assessed at VLV2) were added as auxiliary variables to deal with this issue. These variables assessed the concern of not having enough money to cover current expenses, the need for financial support from someone close to the participant, and the need for welfare. All these three items were coded from 0 for low to 3 for high concerns/needs.

#### 2.3.8. Demographic, Affective and Health Variables

Accounting for previous findings, age, depression and self-rated health were assessed in VLV2 and added to the model. Depression was measured through thirteen items, based on the Wang Self-Assessing Depression scale (SADS [56]) and the General Health Questionnaire (GHQ [57]). The thirteen items were coded from 0 for no feeling of depression to 3 for high feeling of depression. The mean of these items was calculated only if at least six of the items were answered. Current self-rated health was assessed using one open question that asked to indicate one’s current health status from 0 as the worst imaginable health state to 100 as the best imaginable health state [58].

### 2.4. Statistical Analyses

**Figure 1:**
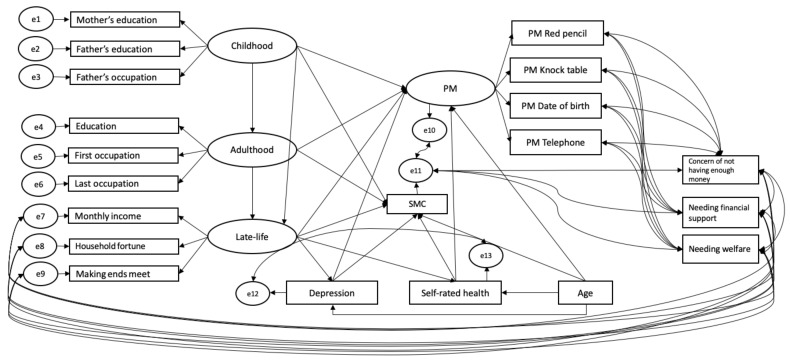
Structural equation model examining the carry-over effect of socioeconomic conditions through the life course and pathways of stages of life course socioeconomic conditions, age, depression and self-rated health on prospective memory (PM) and memory complaints (SMC). Latent variables are represented by ellipses, measured variables by rectangles, and corresponding errors of both latent and measured variables by circles.

A graphic representation of the model tested is shown in Figure 1. We predicted PM performance and SMC from socioeconomic conditions across three life course stages (childhood, adulthood and late-life), while equally considering age, depression and self-rated health in a structural equation modeling framework. PM and socioeconomic conditions at the three life stages were modeled as latent variables. Childhood socioeconomic conditions were indicated by mother’s education, father’s education and father’s occupation; adulthood socioeconomic conditions by the participant’s education and occupations (both first and last); and late-life socioeconomic conditions by their current monthly income, making ends meet and household fortune. To account for a potential carry-over effect of life-long socioeconomic conditions, childhood predicted adulthood and late-life, whereas adulthood predicted late-life socioeconomic conditions.

Late-life socioeconomic conditions and age predicted depression and self-rated health. The residual variances of depression and self-rated health were allowed to covary. Moreover, age, depression and self-rated health predicted PM performance and SMC. Given data for the late-life socioeconomic indicators were not missing completely at random, we followed the procedure suggested by Graham [59] and included three missing-data-relevant variables in the model (“not having enough money to cover current expenses”, “needing financial support from someone close to the participant” and “needing welfare”) and allowed them to covary with the measured variables of the PM latent variable and with the residual variances of late-life socioeconomic indicators and SMC. Because the model contained binary observed variables (the four PM tasks were either failed or succeeded), a robust weighted least square mean and variance adjusted (WLSMV) estimation method was used in Mplus (version 8.3. [60]). We used the chi-square value and corresponding degrees of freedom, the Comparative Fit Index (CFI [61]) and the Root Mean Squared Error of Approximation (RMSEA [62]) to assess goodness of fit (see [63]). Model fit is usually considered good when the ratio between Chi-square and degrees of freedom is smaller than 5 and higher than 2 [64], the CFI is close to 0.95 and when the RMSEA is lower than 0.06.

## 3. Results

**Figure 2:**
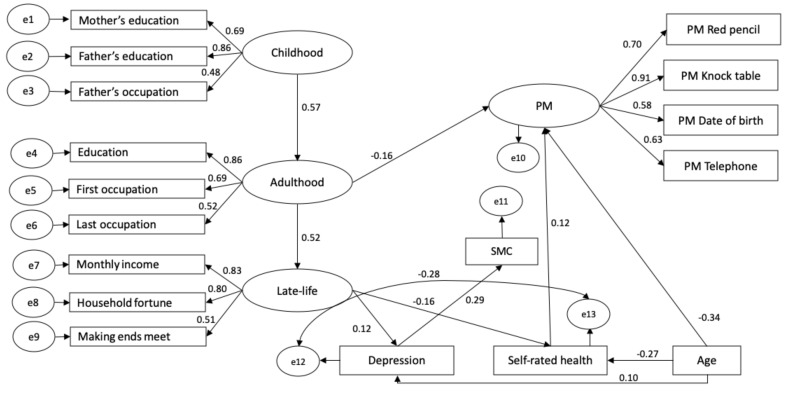
Standardized coefficients estimates for the structural equation model. Non-significant pathways are omitted. For clarity reasons, the missing-data-relevant variables and their significant correlations are not depicted.

Descriptive statistics for the four PM tasks (red pencil, knock table, date of birth and telephone) of the latent PM variable and for the observed variables entered in the model can be found in Table 1 and Table 2. The global fit of the model was good ($\chi^{2}\left(129\right)=392.55,p<0.001;\chi^{2}/df=3.04,CFI=0.91,RMSEA=0.05$). Standardized estimates of significant pathways are depicted in Figure 2. All loadings for the latent variables PM, childhood, adulthood and late-life socioeconomic conditions were significant (see Figure 2 for respective standardized loadings estimates), suggesting a reliable relationship between the factors and their reflexive indicators [65].

### 3.1. Socioeconomic Conditions throughout the Life Course

Childhood socioeconomic conditions significantly predicted adulthood socioeconomic conditions (β=0.57,p<0.001) and adulthood socioeconomic conditions predicted late-life socioeconomic conditions (β=0.52,p<0.001), suggesting a carry-over effect of socioeconomic conditions through the life course. However, the direct pathway from childhood socioeconomic conditions to late-life socioeconomic conditions was not significant (β=0.05,p=0.31).

### 3.2. PM

More disadvantageous socioeconomic conditions in adulthood (β=−0.16,p=0.02) significantly predicted lower PM performance, while childhood (β=−0.08,p=0.19) and late-life (β=−0.08,p=0.13) socioeconomic conditions had no direct concomitant impact on PM. Regarding the demographic, affective and health variables, age (β=−0.34, p<0.001) and self-rated health (β=0.12,p=0.01) predicted PM such that the younger and subjectively healthier the participants were, the better the PM performance was. Depression (β=0.05,p=0.21) did not significantly impact PM.

### 3.3. SMC

SMC was only predicted by depression (β=0.29,p<0.001). None of the effects of age (β=0.03,p=0.33), self-rated health (β=−0.04,p=0.25) or childhood (β=−0.01,p=0.90), adulthood (β=−0.05,p=0.39) and late-life socioeconomic conditions (β=0.001,p=0.98) reached significance. The residual variances of PM and SMC did not correlate (r=0.05,p=0.26), suggesting that these two constructs were not further linked after accounting for the effects of their respective predictors.

### 3.4. Demographic, Affective and Health Variables

Both late-life socioeconomic conditions and age significantly predicted depression (β=0.12,p=0.002 and β=0.10,p=0.003, respectively) and self-rated health (β=−0.16, p<0.001 and β=−0.27,p<0.001). Accordingly, older and socioeconomically disadvantaged participants tended to be more depressed and reported poorer subjective health. The residual variances of self-rated health and depression significantly and negatively correlated (r=−0.28,p<0.001), suggesting that these two constructs were still related beyond the effects of their respective predictors with lower self-rated health relating to higher number of depressive symptoms.

### 3.5. Missing-Data-Relevant Variables

The three missing-data-relevant variables were positively correlated (r=0.58,p<0.001, r=0.50,p<0.001 and r=0.70,p<0.001). Indeed, they significantly correlated with the residual variances of monthly income (r=0.44,p<0.001,r=0.28,p<0.001 and r=0.34,p<0.001, respectively), making ends meet (r=0.55,p<0.001,r=0.28, p<0.001 and r=0.29,p<0.001, respectively) and household fortune (r=0.48,p<0.001, r=0.22,p<0.001 and r=0.28,p<0.001, respectively). However, the three missing-data-relevant variables were not correlated with the measured variables of the PM latent variable and the SMC residual variance (all ps>0.05).

## 4. Discussion

The present study used data from the Vivre-Leben-Vivere study to explore the influence of socioeconomic conditions across the life course on PM (as an objective indicator of everyday memory) and on everyday SMC. Using structural equation modeling, not only the relationships between socioeconomic conditions at different stages of the life course (i.e., in childhood, adulthood and late-life) were assessed, but we also critically investigated the predictive effect of each of these stages on PM and SMC.

### 4.1. Socioeconomic Conditions throughout the Life Course

The analyses revealed that childhood socioeconomic conditions predicted adulthood socioeconomic conditions, and adulthood socioeconomic conditions predicted late-life socioeconomic conditions. Thus, disadvantageous socioeconomic conditions in childhood are passed on to adulthood and those in turn to late-life. However, childhood socioeconomic conditions did not directly predict late-life socioeconomic conditions; this seems to take place only via socioeconomic conditions in adulthood. These carry-over effects support the causal influence of socioeconomic conditions of one stage of the life course on the next stage [19,20] and highlight the importance to not study the effects of different stages of the life course in isolation from the others. Conceptually, this further corresponds with the ideas of cognitive reserve hypothesis, stating that cognitive reserve is built up throughout the lifespan [11,66].

### 4.2. Predictors of PM

Contrary to childhood and late-life socioeconomic conditions, only adulthood socioeconomic conditions significantly predicted PM performance. In line with previous findings on education [42,43], disadvantageous socioeconomic conditions in adulthood predicted lower PM performance. Consistent with the cognitive reserve hypothesis [11,66], advantageous conditions such as high levels of education and occupation seem to build up a buffer against early cognitive decline and thus contribute to preserving cognitive functioning in older age, as was shown with high PM performance in those participants. Disadvantageous socioeconomic conditions, however, seem to prevent building-up this reserve, resulting in lower PM performance in late life. Education in particular was suggested to be vital in preventing cognitive decline due to its influence on occupation [20,66] and since both education and occupation are predictors of cognitive reserve [67,68]. Thus, the finding that only adulthood socioeconomic conditions predict late-life PM performance corresponds with general results on the importance of education in mental functions [13,25,69], and with the impact of occupation on later cognitive functioning [4,13,69].

As brain imaging data were not collected in VLV, neurobiological underpinnings of the obtained effects could not directly be analyzed. However, prior research identified the ventral and dorsal frontoparietal networks, the anterior prefrontal cortex and medial temporal regions as neural correlates of PM [70,71,72] (for overview, see also [73]). Notably, these areas largely overlap with the areas that were identified as being affected by socioeconomic status, comprising effects on both brain structure (e.g., frontal cortex and hippocampus) and function (e.g., prefrontal, frontal, parietal and hippocampal regions) [15]. Hence, the present data suggest that variations in adulthood socioeconomic conditions may be critical for age and individual differences in prefrontal, frontal, parietal and hippocampal regions in the older adult age range, which further is in line with previous studies that have demonstrated effects of education in particular on structural and functional changes in older adults [15,74]. Importantly, although the current study cannot directly test those physiological pathways, obtained behavioral results clearly suggest that these neurobiological effects of adulthood socioeconomic conditions will accordingly show up for PM, where core memory (hippocampal) and attentional control (prefrontal and frontoparietal) networks are interacting and thus the very same brain regions are involved. Moreover, from a clinical neuroscience perspective, PM has been shown to be of crucial importance for several neuropsychological disorders such as Alzheimer’s disease, Parkinson’s disease and Schizophrenia [75,76,77] in which similar brain regions are affected, too. Hence, the present study may serve as basis for future research on both the basic neuroscience and the clinical implications of those socioeconomic context boundaries for cognitive aging.

Socioeconomic conditions in both childhood and late-life do not seem to affect PM performance. Contrary to Aartsen et al. [5] and Ericsson et al. [23], childhood socioeconomic conditions did not predict objective measures of everyday memory. One explanation of that finding might be that the effect of childhood socioeconomic conditions only takes place via their indirect effect on adulthood socioeconomic conditions, as the aforementioned carry-over effects from childhood to adulthood socioeconomic conditions underline. Besides, the results found by Aartsen et al. [5] go in this direction, since the association between childhood socioeconomic conditions and cognitive functioning is smaller when adding adulthood socioeconomic conditions (i.e., education, occupation and financial situation). Supporting this explanation, Ericsson et al. [23] indeed did not include a pathway via adulthood, and thus different conclusions have been drawn compared to the present study. Alternatively, it might be the case that PM in particular, contrary to other cognitive functions, is not affected by socioeconomic conditions in childhood. Given that this was the first study that investigated this effect, replication is required. Furthermore, it may be the case that other childhood factors than the socioeconomic conditions contribute to cognitive performance in later life. Rather than the level of education and the occupation of parents, childhood cognition (see [66]) might rather play a role or whether parents stimulated the child by cognitively challenging activities [12]. Besides, toxic stress or traumatic childhood experiences such as hunger, illness or maltreatment might be stronger predictors of late-life cognition than socioeconomic conditions. Future research is required to investigate these alternative suggestions.

Socioeconomic conditions in late life, namely monthly income, household fortune and making ends meet, did not predict PM performance either. This contrasts prior findings on the role of income for late-life cognition (but note that global cognition measured with the MMSE instead of PM was investigated [8]), raising the question of whether this finding is specific to PM or can be attributed to alternative mechanisms. Adulthood socioeconomic conditions, namely education and occupation, predicted late-life socioeconomic conditions. Therefore, late-life socioeconomic conditions might only ensue from the previous stages of the life course, but, in turn, it might not contribute to PM independently. Conceptually, it seems plausible that later socioeconomic conditions do not affect PM performance, as being exposed to cognitively stimulating environments in that age depends less on socioeconomic conditions than this is the case earlier in life and thus these (mainly financial) conditions might not augment cognitive reserve further. This explanation, however, needs to be further explored in future studies. Beyond socioeconomic conditions, age significantly predicted PM. In line with the literature (e.g., [31,32]), PM performance decreases with increasing age. In addition, self-rated health significantly predicted PM with higher self-rated health predicting higher PM performance. This result supports previous findings that show a positive effect of self-rated health on cognition [78,79].

### 4.3. Predictors of SMC

Contrary to the findings on objective PM performance, SMC were not predicted by socioeconomic conditions at any stage of the life course. First, this highlights that SMC are indeed conceptually different from objective memory performance and thus driven by different mechanisms. This is further corroborated by the observation that the residual variances of PM and SMC do not correlate. Confirming previous findings (e.g., [39]), PM performance and SMC thus seem to not be related in healthy older adults and need to be considered independently.

The observation that none of the socioeconomic conditions at the different life course stages predicted SMC contrasts previous findings that found effects of socioeconomic condition indicators on SMC [45,48]. More precisely, these studies suggested that low socioeconomic status at both childhood [45] and adulthood, particularly education [48], seem to increase the prevalence of SMC. However, the present results do not confirm this finding. Methodological differences might contribute to these contrasting results. While participants of the present sample were on average 80 years old, participants of the other studies were much younger (on average 17 years younger in the study by Kliegel and Zimprich [48] and 60% of the sample of Nishizawa et al. [45] was younger than 75 years). Furthermore, key variables were operationalized differently, such as a more detailed assessment of SMC by both Kliegel and Zimprich [48] and Nishizawa et al. [45], but a less objective assessment of childhood socioeconomic conditions in the latter (i.e., subjective rating of social status when 15 years old) and the usage of school years instead of the highest participant’s educational degree in Kliegel and Zimprich [48]. A more systematic exploration of these methodological differences might shed light on the relationship of socioeconomic conditions across the life course on SMC.

Among all predictors of the present analysis, only depression predicts SMC with more feelings of depression also increasing SMC. This corresponds with findings suggesting that SMC are predicted by personality and affective variables, namely neuroticism, depression, anxiety, age-related stereotypes and subjective age [39,47,48,49,50]. Indeed, these variables might contribute more strongly to SMC than to objective memory performance. Alternative explanations of our results might be related to the differing types of variables of the outcomes: PM was entered in the model as a latent variable with four measured variables, whereas SMC was only one item forming an observed variable. This explanation may also correspond with the result that neither age nor self-rated health predicted SMC, again contrary to previous findings on age [36,80] and self-rated health [80,81]. Reflecting SMC by more than one item could augment the validity of the measure. Clearly, future studies need to further explore predictors of SMC, preferably by constructing SMC in a latent way using several items.

### 4.4. Late-Life Socioeconomic Conditions and Age Predictors of Self-Rated Health and Depression

Finally, we showed that late-life socioeconomic conditions significantly predicted self-rated health and depression, as also demonstrated in the literature [82,83,84]. Specifically, disadvantageous socioeconomic conditions in late-life predicted lower self-rated health and more feelings of depression. Interestingly, self-rated health, in turn, influenced PM, while depression influenced SMC. This could indicate an indirect link of late-life socioeconomic conditions on both PM and SMC via the route of self-rated health and depression, respectively (see [48] for similar results).

However, as the present data are cross-sectional, a reverse causation cannot be excluded and needs to be ruled out by more longitudinal research. Moreover, in line with the literature, older participants generally show lower self-rated health [85,86] and higher levels of depression [87] (but see also [88]).

### 4.5. Limitations and Future Research

As many of the measures that were used as either predictor or dependent variable were assessed at the same timepoint, reverse causation of some of our results cannot be excluded and limits the interpretations of our findings. However, this mainly concerns the variables depression, self-rated health and socioeconomic conditions in late-life, which were assessed at the same time as PM and SMC. Socioeconomic conditions over the life course, importantly, referred to information from years preceding the assessment, which justifies assuming a causal influence from one stage of the life course to the next one. However, variables of socioeconomic conditions were self-reported and assessed mainly retrospectively, therefore potentially being subject to social bias and recall bias. Moreover, as main outcomes were measured in the second wave, a selectivity bias could affect results, because the remaining participants are potentially the ones with higher cognitive performances in the first wave. In addition, the fact that the location was not the same across all participants and that prospective memory tasks were less standardized than in computerized laboratory studies due to the VLV study design might be a limitation. On the contrary, the setting of the VLV study had the advantage of having access to a large and representative part of the aging population in Switzerland, particularly to older participants who may be less mobile or less familiarized with the use of a computer. Furthermore, one might question why the latent constructs of socioeconomic conditions were constructed as reflexive and not as formative. Even if it is possible to construct this model in a formative way, we decided on the reflexive way as we were interested in the variances shared between the measured variables and not by the specific contribution of each indicator to the latent construct. Moreover, this choice was made to facilitate comparability across studies [65].

As indicated above, future research is required to investigate SMC at the latent level. Furthermore, as the present study only assessed event-based prospective memory and not time-based prospective memory, further studies are needed to extend the present results to time-based prospective memory. The use of a longitudinal design is necessary to truly assess change, particularly to account for the dynamic effects of socioeconomic conditions across the life course on PM and SMC. Moreover, as only adulthood socioeconomic conditions are found to predict PM performance, further research needs to focus on the mechanisms underlying these effects. Finally, this study focused on cognitive reserve hypothesis, however, structural and functional brain differences, variations of cognitive functions at baseline, as well as diverging behaviors and lifestyle distinctions between individuals with high and low socioeconomic conditions may explain differences in late-life cognitive performance and/or cognitive decline in older age [15,89]. Hence, further studies including brain imaging data are needed to explore these effects. In addition, it will be important that future studies replicate these effects in an experimental standardized design, use several items to assess SMC and extend results to time-based prospective memory.

### 4.6. Conclusions

The present analyses highlight a carry-over effect of socioeconomic conditions from each stage of the life course to the next one. Disentangling the specific effect of each stage, only socioeconomic conditions in adulthood predicted PM performance as an objective measure of everyday memory in older adults, but not socioeconomic conditions at childhood or later life. In contrast, SMC were not predicted by socioeconomic conditions at any stage. The findings support previous research suggesting that SMC are not predicted by the same factors as objective memory measures, but rather by affective variables such as depression. This study highlights and alerts policies to the importance of the influence of the previous stage of socioeconomic conditions on the actual stage and the role of adulthood socioeconomic conditions in successful aging.

## Figures and Tables

**Table 1:** Frequency table of the observed PM variables entered in the model.

PM Tasks	Task Success	Number of Observations	Percentages
Red Pencil			
	Failure	447	45.5%
	Success	535	54.5%
Knock table			
	Failure	649	66.3 %
	Success	330	33.7%
Date of birth			
	Failure	741	75.5%
	Success	241	24.5%
Telephone			
	Failure	669	67.4%
	Success	324	32.6%

**Table 2:** Descriptive statistics of the observed variables entered in the model.

Variables	N	M	SD	Range
Age	993	80.56	6.56	70.21–102.56
Self-rated health	895	75.96	17.47	0–100
Depression	967	0.74	0.35	0–2.31
Father’s occupation	932	4.89	2.70	0–10
Mother’s education	945	3.77	1.40	0–6
Father’s education	937	2.98	1.62	0–6
First occupation	985	5.83	1.70	0–10
Last occupation	975	4.85	2.50	0–10
Education	979	2.07	1.41	0–5
Monthly income	918	3.78	1.86	0–8
Making ends meet	961	0.71	0.78	0–3
Household fortune	863	2.31	1.53	0–5
Concern of not having enough money	958	0.39	0.74	0–3
Needing financial support	956	0.34	0.81	0–3
Needing welfare	951	0.38	0.85	0–3
Memory complaints	965	1.17	0.57	0–3

## Data Availability

The data presented in this study derived from VLV1 are openly available at the Swiss Centre of Expertise in the Social Sciences (FORS), https://forsbase.unil.ch/project/study-public-overview/14791/0, project reference number 12941. Data that is derived from VLV2 is currently prepared for being openly available in FORS, too. Until then, these data are available on justified request from the corresponding author.

## Abbreviations

The following abbreviations are used in this manuscript:

PM	Prospective Memory
SMC	Subjective Memory Complaints
MMSE	Mini-Mental State Examination
